# Development and evaluation of a novel 3D simulation software for modelling wood stacks

**DOI:** 10.1371/journal.pone.0264414

**Published:** 2022-03-16

**Authors:** Felipe de Miguel-Díez, Philippe Guigue, Tim Pettenkofer, Eduardo Tolosana-Esteban, Thomas Purfürst, Tobias Cremer

**Affiliations:** 1 Department of Forest Utilization and Timber Markets, Eberswalde University for Sustainable Development, Eberswalde, Brandenburg, Germany; 2 Chair of Forest Operations, University of Freiburg, Freiburg, Baden-Württemberg, Germany; 3 Dr. Philippe Guigue Software Artisan, Berlin, Germany; 4 Arbeitsgemeinschaft Rohholz e.V., Berlin, Germany; 5 E.T.S.I. Montes, Forestal y del Medio Natural, Universidad Politécnica de Madrid, Madrid, Spain; USDA Forest Service, UNITED STATES

## Abstract

Assessing the solid wood content is crucial when acquiring stacked roundwood. A frequently used method for this is to multiply determined conversion factors by the measured gross volume. However, the conversion factors are influenced by several log and stack parameters. Although these parameters have been identified and studied, their individual influence has not yet been analyzed using a broad statistical basis. This is due to the considerable financial resources that the data collection entails. To overcome this shortcoming, a 3D-simulation model was developed. It generates virtual wood stacks of randomized composition based on one individual data set of logs, which may be real or defined by the user. In this study, the development and evaluation of the simulation model are presented. The model was evaluated by conducting a sensitivity and a quantitative analysis of the simulation outcomes based on real measurements of 405 logs of Norway spruce and 20 stacks constituted with these. The results of the simulation outcomes revealed a small overestimation of the net volume of real stacks: by 1.2% for net volume over bark and by 3.2% for net volume under bark. Furthermore, according to the calculated mean bias error (MBE), the model underestimates the gross volume by 0.02%. In addition, the results of the sensitivity analysis confirmed the capability of the model to adequately consider variations in the input parameters and to provide reliable outcomes.

## Introduction

In many countries, industrial wood is generally commercialized in stacks. Concerning this process there are three important reference values: gross volume of the wood stack, solid wood content (or net volume) and the conversion factors that relate to each other.

To determine the gross volume of the stack, several methods have been developed over time. The most common of these is the sectional volumetric measurement method. This method is subject to numerous variations depending on the country in terms of the measurement of the stack height or the section length [1]. Currently, there is no uniform, standardized stack volume measurement method for the European Union. However, European standards recognize the round timber measurement and volume calculation rules of several European countries; for instance, the method outlined in the German framework for round wood trade (RVR) [2]. When measuring according to the method included in the RVR, the stack is, depending on its exact length, divided into equal-length sections. The last section of both stack sides (front/rear) is usually shorter since it covers the remaining length to the stack end. The first sections form the stack part A and the last section is known as stack part B. The height is measured at the center of each section and the arithmetic mean value is subsequently computed. The gross volume is calculated by adding up the volume of stack parts A and B. To calculate the volume of stack part A, the average height of the stack, the stack length and the stack width (which corresponds to the length of the logs) are multiplied. The volume of stack part B is the product of the length of the logs, the height of the stack in the middle of this part and the length of this part [3, 4]. In the present study, the gross volume is expressed as stacked cubic volume in m^3^ (st) since this unit is more widely accepted.

With the development of new technologies and their implementation in the forest sector, an additional method to measure the gross volume of a stack emerged: the photo-optical method [1, 5]. Although functioning and algorithms of the different applications can vary to a greater or lesser extent, nearly all of them provide the most relevant parameters for the commercialization process of round wood: gross and net volume as well as the conversion factors.

The net volume of a stack, also known as the solid wood content, is the amount of wood in a stack. There are several methods to determine the solid wood content. The most accurate is the water displacement method [6, 7]. Another traditional method consists of measuring the log diameters on both ends inside the bark and calculating the volume by applying a formula [7, 8]. However, the application of conversion factors is frequently used, which involves multiplying these by the gross volume.

Such conversion factors between gross and net volumes have been applied for several decades in relation to the assortment. The conversion factors are dimensionless parameters that range between 0 and 1. Usually, the values of the conversion factors range between 0.6 and 0.7, and in extreme cases, between 0.5. and 0.8 [9]. The conversion factors can be determined *in situ* by applying the quadrant method, e.g., in Ireland [10]. Another method involves computing a final factor by adding up or subtracting points from an initial specific value for each tree species according to visual assessment of several influencing parameters such as mean diameter, bark presence, length, etc., as used in Sweden [11]. In addition, the conversion factors can also be predefined based on statistical databases for a given wood assortment. These have been determined in previous research efforts, e.g., in Romania [12], and they are included in the measurement guidelines of numerous countries such as Austria [13] and Poland [14].

Both conversion factors and stack volume are influenced by several parameters that have been identified and analyzed since the end of the 19^th^ century [15, 16]. The influencing parameters have been analyzed in several research efforts mainly in recent decades in Germany and Romania [12, 17–19]. However, the influence that all logs and stack parameters have on the stack volume, and in turn on the conversion factors, has not yet been analyzed individually. Moreover, previous research studies about this topic have lacked a broad statistical basis since the conversion factors were determined based on a low number of stacks. The most important reason for this limitation is the high cost of collecting enough data to ground those analyses.

Therefore, it was necessary to find a solution for analyzing the influencing parameters on the stack volume and conversion factors on a broad statistical basis. In previous studies in the same field, simulation software was successfully developed and used. For instance, in 2017, a simulation model for measuring the volume of wood stacks was demonstrated. The authors accomplished the development of an algorithm which allowed for measurement of the gross volume of a stack by modelling the logs from pictures of real stacks [20]. Furthermore, another similar simulation model was recently developed. In this case, the influence of some log parameters on the conversion factors could be investigated using this simulation model on a broad statistical basis. However, even though the simulation outcomes of this model were somewhat in line with the results from previous research, the model was not validated with real measurements and was not able to virtually generate the logs in detail, e.g., it could not simulate the bark [21]. Considering the numerous advantages of implementing IT-technologies, including cost-effectiveness, the approach of developing and using a simulation software was chosen for this study.

Holzworth *et al*. [22] stated that before a model may be used for its given purpose, it is necessary to evaluate its reliability and practicality so that the user can be confident that all simulation outcomes will be valid. Prisley and Mortimer [23], who first stated a lack of a universal approach for evaluating a model, afterwards compiled three different methods to carry out the evaluation: 1) peer-review, 2) quantitative analysis of model results and 3) sensitivity analysis. In the present study the last two approaches were applied. In the same literature review, Prisley and Mortimer [23] described the quantitative analysis as the most familiar view of model validation, which consists of a quantitative comparison of predicted values against observed data. For this purpose, numerous statistical methods were applied from visualization comparisons to regression analysis. However, whereas this method is extensively accepted as an essential part of the model evaluation, there is no global statistical technique recognized to implement it. Concerning the third approach, the American Society for Testing Materials (ASTM) in 1992 defined this as the examination of the degree to which the model output is influenced by modifications of determined input parameters [23]. Prisley and Mortimer confirmed the frequent use of this approach [23]. For instance, this analysis was implemented by Holzworth *et al*. [22] as a series of tests which contrasted simulation results with simple relationships from the literature.

The first objective of this study was to resolve the shortcomings of manual data collection by using modern technology to obtain large databases to carry out statistical analyses. Hence, the study aimed to develop a 3D-simulation software: HOPOSIM (German contraction for wood stack simulator). The second objective of this study was to evaluate the developed simulation model by means of 1) a quantitative analysis of the model results comparing these with real measurements of wood stacks and 2) a sensitivity analysis conducted by modifying the lengths of the logs and comparing the resulting conversion factors with others gleaned from the literature.

## Material and methods

### Software development

The simulation software is composed of two distinct parts: an application dedicated to data collection and preparation and a simulator responsible for the 3D modeling and the actual 3D wood log stack physical simulation.

The data preparation software consists of a desktop Windows-based application developed using the.NET Framework. The data model was implemented in C# and the Windows Presentation Foundation (WPF) was used as a framework for designing its user interface (UI). The software defines and generates the data necessary for the 3D physical simulation of the wood stack. These data include the parameterization of each log, the definition of the geometry of the stack (i.e., the desired shape of the stack), its quality (proportion and initial average angle of the ‘cross’ logs) as well as the specific physical attributes of the tree species. All configuration data is persisted into a SQLite relational database by taking advantage of the Entity Framework (EF) core data access technology.

**Fig 1:**
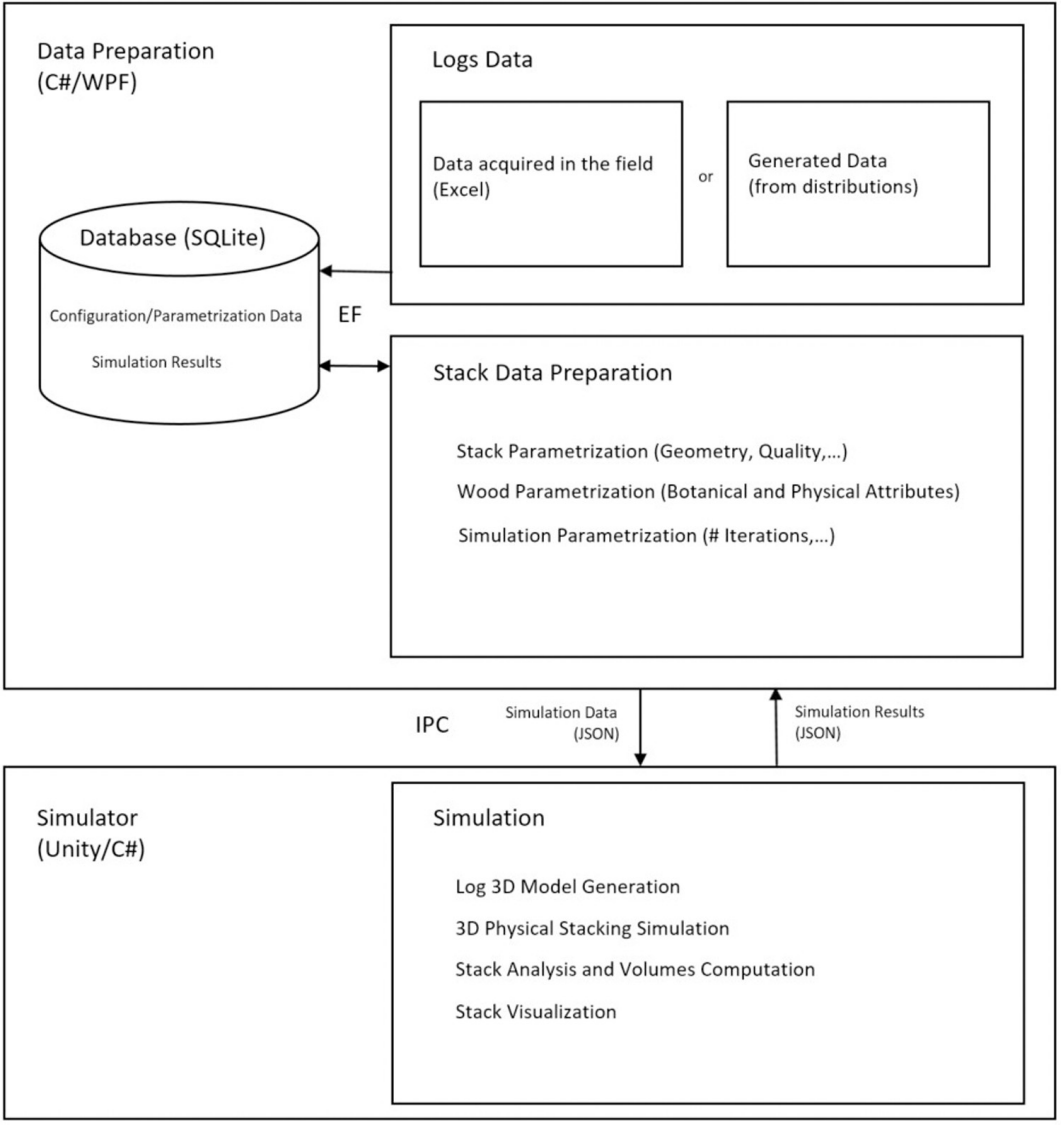
Flowchart of the simulation process and the software used.

The simulation process itself proceeds in two steps. The first stage generates 3D models of the wood logs and the second stage stacks these logs using a virtual environment that incorporates laws from the physical world. An overview is depicted in Fig 1.

The simulator is based on the Unity software, which supported the geometric modelling and computation of the logs by providing the necessary data structures and geometrical operations, while its game and physics engines powered the complex physical simulation processes running during stacking simulations. The built-in Unity render pipeline was adopted to obtain a realistic render of the logs by exploiting the materials and textures assigned to the bark and sections of the logs. In addition to those used for the general simulation logic, custom C# scripts were implemented for the computation and display as overlays of the hulls, sections and log numbers as well as to provide camera controls for intuitive navigation of the scene in visualization mode.

The inter process communication (IPC) mechanism, which allows the data preparation and simulation processes to communicate with each other and synchronize their actions, was implemented with the help of Windows Sockets. The data exchanged between the two processes is in JavaScript Object Notation (JSON) format.

### Selection of the simulation parameters and model assumptions

Based on the results of a comprehensive literature review, which encompassed previous research works and the measurement guidelines of numerous countries, e.g., Sweden [11], Ireland [10], the United Kingdom [24] and Germany [3], several log and stack parameters were selected as input parameters.

The selected parameters were classified into two groups: log parameters and stack parameters. The log parameters were length; diameters at butt, midpoint and top of the log; taper; crookedness; percentage of presence of a determined crook form in the stack; ovality; buttressing with regard to the number of butt swells and their radii; bark damages; wood density; and delimbing quality (which includes the percentage of logs with protruding branch stubs, the dimension of the branch stubs and their percentage of presence in each log).

The stack parameters were the friction between the logs; minimum length of the stack; average angle of the stack´s sides; stacking direction of the logs; and stacking quality, which was defined as a determined percentage of stacked logs with a given deviation from the longitudinal stacking axis and is expressed in degrees. Moreover, it is possible to simulate stacks within a saddle constituted by two lower logs as well as to let the model virtually stack the logs with a randomized stacking direction.

In order to facilitate the introduction of specific characteristics of some tree species, three determined tree species Scots pine (*Pinus sylvestris* L.), Norway spruce (*Picea abies* (L.) H. Karst) and European beech *(Fagus sylvatica* L.) can be parameterized in advance in relation to buttressing and delimbing quality. Afterwards, the tree species can be selected when introducing the input parameters meaning that this additional information does not need to be repeatedly introduced. In addition, there is another option to parameterize any tree species.

To model the virtual logs in a sufficiently realistic way, several assumptions about the logs´ characteristics were made.

**Fig 2:**
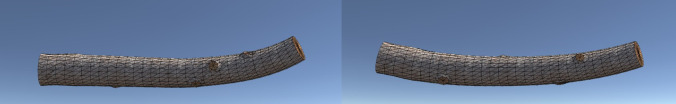
1^st^ crook form (left) and 2^nd^ crook form (right) assumed in the simulation model development.

Due to the broad spectrum of crook forms, e.g., concerning the tree species Norway spruce [25], two specific shapes were selected in addition to a straight type (Fig 2). This decision was based on the high frequency at which these shapes appeared in the logs when measuring the input parameters. The degree of crookedness corresponds to the Euclidian distance measured from the levelling staff set on the crooked side to the log at the deepest point. In addition, it was assumed that no log with crotch was simulated.

**Fig 3:**
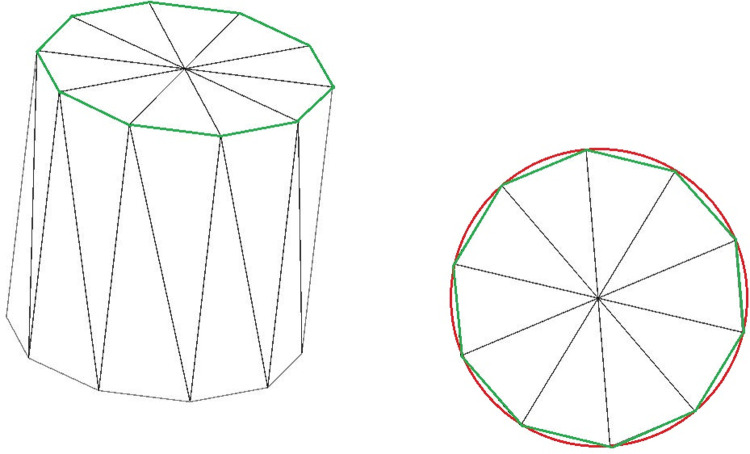
Difference between real (red) and simulated log shape (green).

The simulation model considers the bark damages as the proportional reduction in the bark thickness of the logs from 0% to 100% (0 mm). This was assumed in order to simplify the location of damaged or missing bark in the log and the degree of damage to the log. The virtual representation of each log is based on triangle meshes that only provide an approximation of the real log’s shape (Fig 2). Such an approximation is inherent to the discrete representation of 3D shapes and is illustrated in Fig 3 where the red circle represents a section of the real ideal log, and the green polygon shows the shape of the modelled log for the same section. For the real simulation run, the size of the triangles used in the mesh is reduced (more sides are added to the green shape) which minimizes the approximation error.

In theory, this approximation does not produce a significant difference. When comparing the volume of 500 simulated cylinders with HOPOSIM and the volume of the same cylinders calculated with mathematical formulae, the difference was 0.11%. Based on this marginal difference, it was assumed that the volume of the simulated logs accurately approximates the volume of the real ones.

A further assumption relates to the stacking process. In principle, it is assumed that every log is stacked in a spatially parallel way. The deviation in degrees of a determined percentage of stacked logs can be defined by the user. Both stack ends are evened so that no log end deviates more than 7.5 cm from the average end surface of the stack. The stack must present a trapezoid form. Each stack is built with logs of a single tree species and a sole product length. No stack with curved form is simulated.

Finally, the last assumption concerns the parameter friction. This parameter is defined as the resistance to relative displacement between two surfaces and depends on the specific features of the interacting surfaces. It is quantified through the friction force, which delays the displacement of an object with respect to another; in this study, of a log with respect to the other logs in the stack. The magnitude of the friction force corresponds to the product of the coefficient of friction, which is a dimensionless parameter, and the resulting normal force. The resulting normal force is due to the proper weight of the logs and the external forces acting on the system [26].

The Unity libraries consider both dynamic and static friction coefficients. However, only the mean value of the coefficient of dynamic friction and the coefficient of static friction can be introduced in the model [27].

As no reference relating to the dynamic and static friction coefficients for Norway spruce bark could be found, a range of values from 0.5 to 0.7 was determined based on values and results of previous studies which considered different tree species, wood surfaces and logs with bark, debarked, partially debarked and watered [26, 28, 29]. After that, several simulations were run introducing different values within that range. The resulting mean values of the simulated gross volumes for 0.5 and 0.7 ranged from 49.8 to 50.6 m^3^(st). The variation was 0.8 m^3^ (st) or 1.6% and considered small. Under these circumstances, a decision was made to take the value 0.6 for this study, since this number is the mean value of the determined range between 0.5 and 0.7.

### Model evaluation

The simulation model was evaluated based on real measurements of logs and stacks constituted by those logs. The real measurement results were introduced in the model as input parameters. The input parameters required to perform the simulations were measured in 405 logs of Norway spruce (*Picea abies* L.) and 20 stacks. The stacks were randomly built by a forwarder after measuring the logs. In doing so, the most similar virtual reproduction of the real stacks was ensured. The real stacks were measured in accordance with the RVR method described earlier [4].

The logs were prepared in the forestry district of Chorin (52° 53’ 22” N, 13° 52’ 06” E), located in Brandenburg, Germany. The age class of the stand was from 40 to 60 years. Their average length was 250 cm, ranging from 227 to 268 cm, while their mean diameter was 206 mm, ranging from 145 to 315 mm. The average tapering was 10 mm/m, ranging from 0 to 47 mm/m and the average crookedness was 2 mm/m, ranging from 0 mm/m to 27 mm/m. In principle, the logs belonged to an assortment destined for manufacture pallets. However, the logs complied with all industrial wood requirements and were classified accordingly as industrial wood. The evaluation of the model was carried out in summer.

As input data, the required log parameters were measured in each log with a tree caliper, a tape, a levelling staff, folding rule, and a Swedish bark gauge. The measurement method of the log parameters was identical to that applied by De Miguel-Díez *et al*. [30].

To enable an accurate comparison of the initial net volume of the stack over and under bark, the volumes of the real logs with bark were measured by means of a xylometer. However, it was also necessary to assess each log´s bark volume in order to calculate the net volume of the stack without bark and to deduce the conversion factors. To achieve this aim, the bark volume of 40 randomly selected logs, was quantified. After being measured using the xylometer, the logs were debarked manually and immersed again. The difference between the first and the second measurements corresponded to the bark volume of each log. The resulting average bark volume of these 40 logs was extrapolated to the 405 logs which constituted the stacks. From this computed value, it was possible to determine the net volume of all logs under bark, and thus of the stack, by subtracting the bark volume from the sum of the volume of every log.

To estimate bark damage, the perimeter of 40 logs chosen at random was measured with a tape at ten points every 25 cm along the log. Measurement was taken in cm to one decimal place. In doing so, the part of the perimeter without bark was measured, and its proportion could be calculated as a percentage of bark damaged at that measurement point. The percentage of damaged bark of the whole log was obtained by calculating the average value of the resulting percentages at each of the ten measurement points. In turn, the mean value of bark damage corresponding to those 40 logs was calculated and the result was extrapolated for the 405 logs, thus obtaining the percentage of bark damage in the whole stack. This measurement was performed before and after the logs were stacked. It must be pointed out that bark loss occurred predominantly from building the 11^th^ to the 18^th^ stack. As such, the gross volume of the real stacks varied slightly from the 1^st^ to the 10^th^ stack and from the 19^th^ to the last stack, whereas the gross volume of the real stacks varied more from the 11^th^ to the 18^th^ stack.

Further input parameters were measured and introduced in the model. The average angle of every stack side was 40°. The raw wood density was 621 kg/m^3^. The percentage of logs with the small end stacked forwards was 53%. The minimum stack length introduced was 10 m. The percentage of logs with a crook form corresponding to the 2^nd^ form in Fig 2 was 60%. The delimbing quality was poor, with a percentage of logs with protruding branch stubs of 70%.

Based on the aforementioned differences in bark loss, the analysis was conducted by comparing the gross volumes of the first 10 real stacks and the last 10 real stacks with 50 runs of simulations for each bark damage percentage, i.e., the measured values before and after staking the logs. This means that from each simulation run, 10 modelled stacks resulted. In doing so, a representative number of 1000 total modelled stacks was obtained. The selection of those real stacks was based on the fact that the bark loss was marginal when they were formed consecutively. To simplify this analysis, only the gross and net volumes over bark and under bark that were measured according to the RVR method were considered.

From the measured data, the length of the real logs was modified in a discretionary way. After that, three simulation runs with 500 iterations for each modified length were carried out in order to test the simulation model´s capability to take this variation into consideration and provide credible results. Concretely, the length of the logs was modified to 1, 2 and 3 m and the resulting average conversion factors were compared with conversion factors for these assortments, which are used in the round timber measurement and volume calculation rules of several countries. With the aim of obtaining representative results, a decision was made to simulate 500 stacks for each length, with the assumption that this number was high enough to provide a wide statistical basis.

Once the simulation outcomes were obtained, the analysis was performed in three steps. Firstly, the basic descriptive statistics were computed for the considered real and simulated stacks depending on the different bark damage percentages. Subsequently, the differences between those values related to the gross and net volumes of the real and simulated stacks were compared. Next, the root mean square error (RMSE) [31] was calculated from the real stack volumes and the simulated stack volumes of 50 simulation runs considering different bark damages before and after stacking in order to determine the deviation magnitude. Afterwards, the mean bias error (MBE) [32] was calculated from the same values. The MBE calculation was carried out in order to obtain a second reference value to evaluate this model. In addition, the result of the MBE indicates with its sign whether the model systematically overestimates or underestimates the predicted values in comparison with the measured stack volumes. The evaluation was conducted using the program RStudio (version 1.4.1103) developed by RStudio, Inc. [33]. To visualize the simulation results, the package “*ggplot 2”* was used [34]. The calculation of the RMSE and MBE was conducted using the package “*tdr”* [35].

## Results

### Software interface

**Fig 4:**
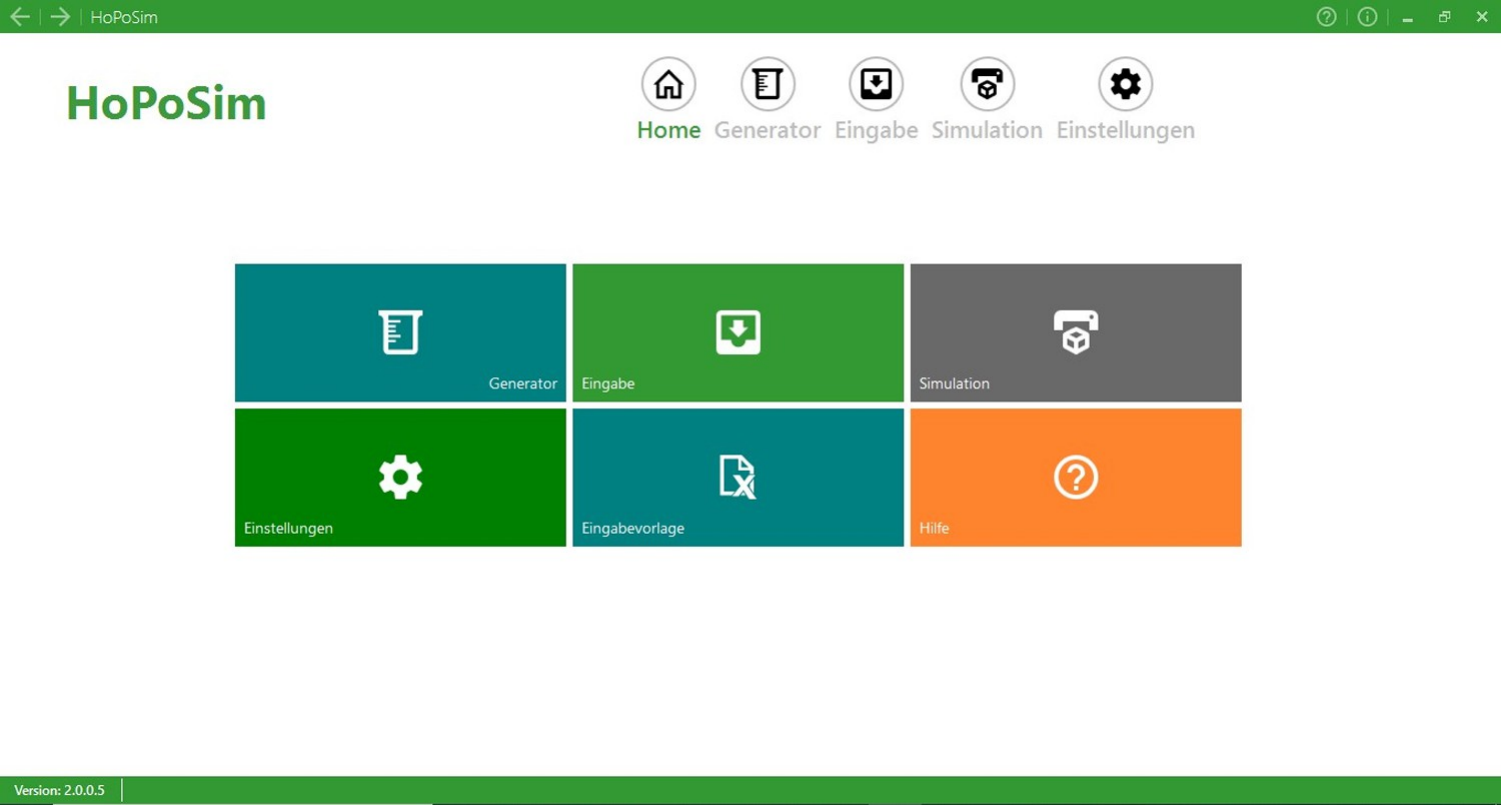
Main interface of the software.

The software presents a main interface in German from which it is possible to navigate to the various software modules (Fig 4).

These are: the generator (Generator) for producing a datasheet of virtual logs with specified characteristics; the data input section (Eingabe) to import a datasheet from real measurements or with data defined by the user at his discretion; and the simulator (Simulation), where the simulation of wood stacks is performed and the output of data is generated. In this interface, the simulation can be started and stopped, and the outcomes can be exported in a table in Excel format. Further parameters related to the simulation can also be selected in this part. These are the number of stacks that will be simulated and the number of support points per meter that will be applied when reproducing the measurement of the modelled stack with the photo-optical method. After starting the simulation, a new window emerges in which the simulated stacking procedure can be visualized.

The simulation results are:

- Gross volume of the stack measured in accordance with the sectional volumetric method included in the RVR.
- Gross volume measured by virtually reproducing the use of the photo-optical method.
- Gross volume computed by measuring the surfaces of both stack sides and multiplying these measurements expressed in m^2^ by the length of the logs.
- Net volume of the stack under and over bark.
- Conversion factors that relate to the aforementioned outcomes.
- Percentage of bark volume in the stack

It is also possible to customize general settings of the application (Einstellungen), such as the path to the 3D generator, the directory to save the results or modify the parameterization of tree species and stacking quality. Lastly, in the help section (Hilfe), a comprehensive guide in English for using the software can be read.

### Simulation process

The simulation software is composed of two parts: an application dedicated to data collection and preparation, and a simulator responsible for the 3D modelling and the actual 3D wood log stack physical simulation. Once all these parameters have been assigned, the data can be prepared for input to HOPOSIM3D, the 3D simulation application. The parameterization of the wood logs is carried out either by importing data acquired in the field, or by generating these data automatically from the distributions of the relevant parameters.

**Fig 5:**
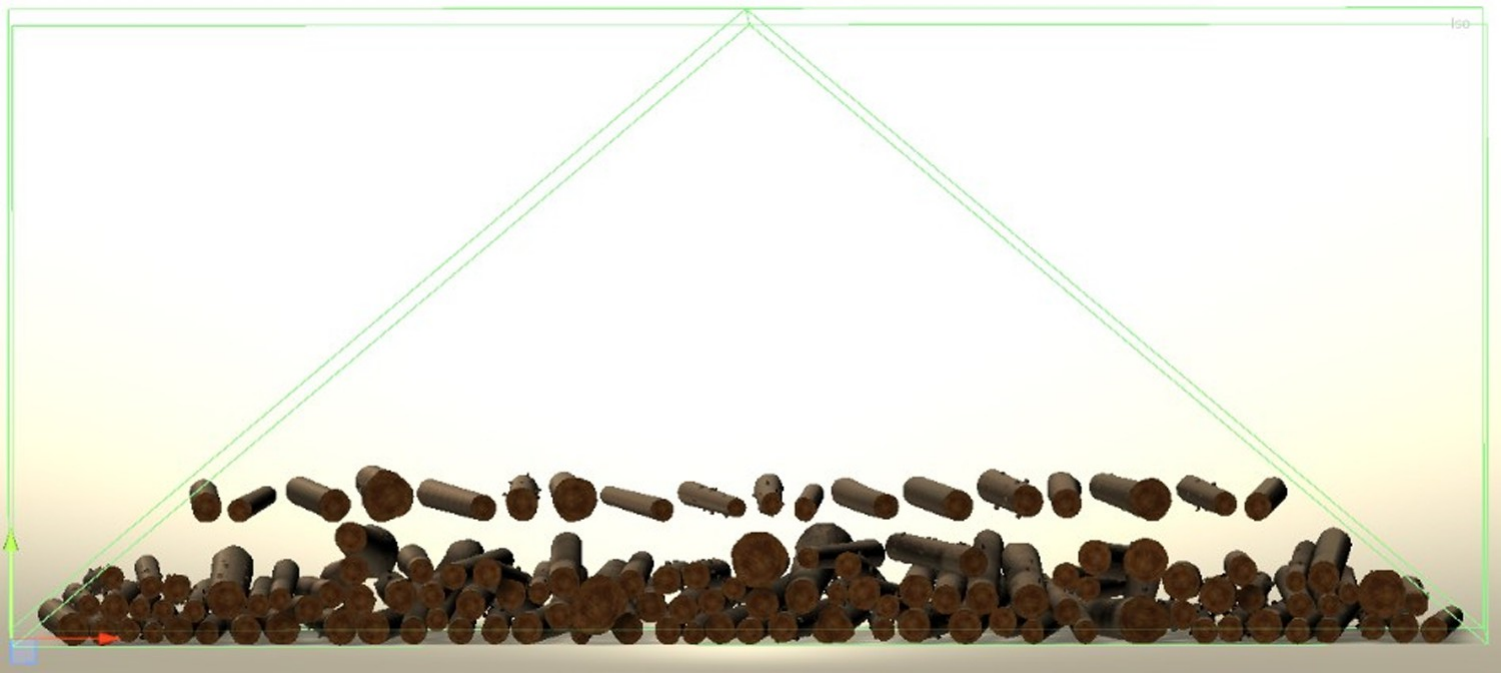
Virtual stacking process.

From the different parameters characterizing a given log, HOPOSIM3D generates a 3D triangle mesh (Fig 2). The modeling algorithm generates convex triangle meshes based on the log specifications. These meshes are used by the physics engine for collision detection and for rendering. In this last case, textures are mapped to the 3D models. Once each trunk has been modeled, the stacking algorithm proceeds iteratively by positioning the logs in turn in horizontal layers on top of the stack under construction (Fig 5).

The position of each log in a layer is constrained to lie in the triangular prism defined by the length, depth and side angle of the stack provided by the user. The logs in each layer are initially positioned with respect to their bounding box (i.e., the smallest axis aligned enclosing box of each log’s mesh points). Once the layer has been completed, each log belonging to the layer is then submitted to the physics engine integrated in the Unity software (the Nvidia PhysX engine).

The physics engine makes it possible to simulate physics during the whole stacking process to ensure that the logs behave correctly and respond to collisions (according to the density, friction and other physical properties set by the user) and gravity as they exist in the real world. The algorithm thus proceeds by constructing successive layers of logs until all the input logs are processed.

Once all of the logs have been stacked and have reached a stable configuration, the algorithm performs the stack analysis by calculating the different areas for the stack’s front and back sides, and its derived volumes. HOPOSIM3D reproduces accurately the execution of three measurement methods to calculate the stacked cubic volume: the RVR method [3, 4], the photo-optical method and the measurement of the surface of the stack´s front and back sides, being multiplied by the average length of the logs or by the order length of the logs.

**Fig 6:**
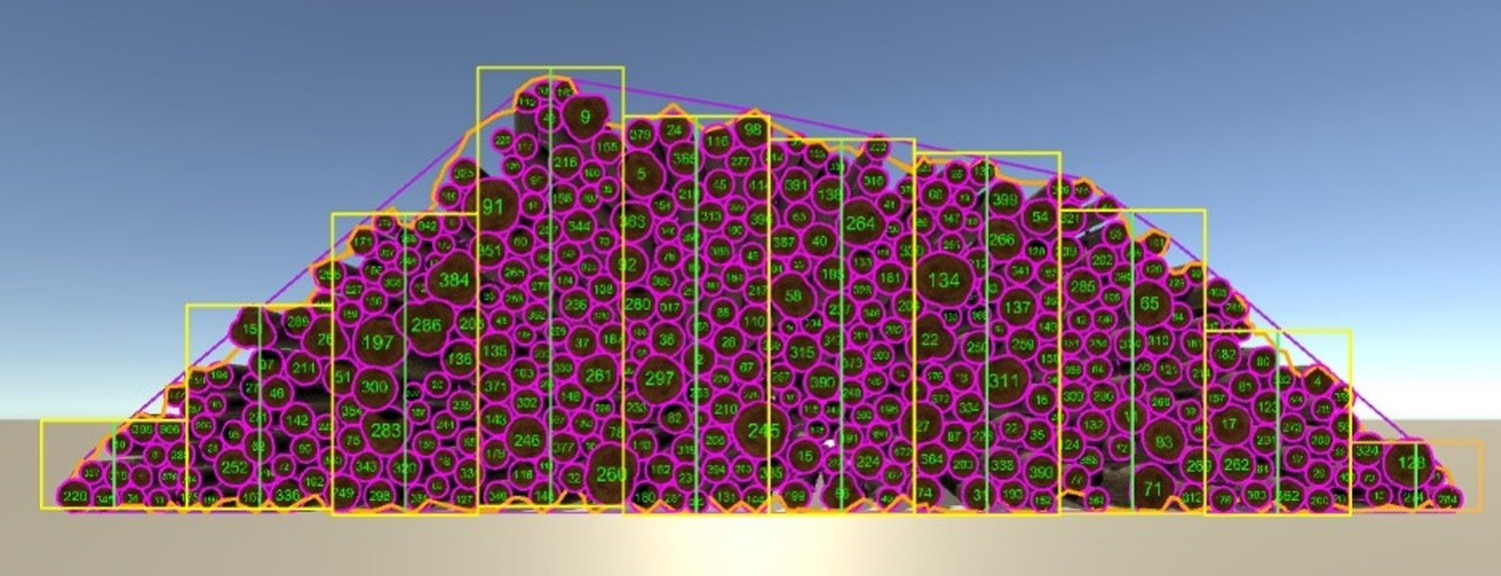
Virtual reproduction of the different measurement methods as well as numeration of logs.

The virtual reproduction of the aforementioned measurement methods is represented in Fig 6 where the yellow sections (and in orange the stack part B) correspond to the virtual reproduction of the RVR method, the orange polyline measures the surface of the stack´s sides and the purple polyline virtually reproduces the photo-optical method. In addition, each log is numbered, and the surface of both ends marked in purple.

The net volume is finally calculated by adding up the volume of each wood log. The latter is calculated by adding the signed volume of its tetrahedron (formed by the triangle and topped off at the origin of the 3D coordinate system) for each triangle composing the log mesh.

The conversion factors can be derived from these values and are communicated back to the HOPOSIM application for data aggregation.

Such a simulation is carried out as many times as the user specifies. For each iteration, the order of insertion of the 3D log models is randomly shuffled to ensure variations in the stacking results.

Once all such iterations have been processed, the application generates a report integrating the mean distribution of the input log parameters (tapering, crookedness, ovality,. . .) as well as the different calculated measurements (area, volume and conversion factors) for analysis.

**Fig 7:**
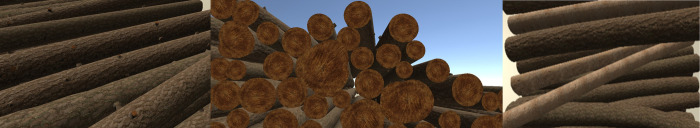
Simulation details: Branch stubs, butt swells, debarked logs (from left to right).

Notably, HOPOSIM3D virtually generates the logs in detail. These virtual replicas correspond to specific characteristics of different tree species such as the presence of butt swells in Norway spruce or of branch stubs protruding when the delimbing quality is not good. When a simulated log has no bark (value of bark thickness is 0), it appears with a clearer color as it might be found in the natural environment. Fig 7 shows the branch stubs, the butt swells and some debarked logs.

### Exemplary model evaluation

The basic descriptive statistics are displayed in Table 1. The simulation model overestimates the average gross volume of the real stacks by 0.2 m^3^ (st) or 0.4% for 21% bark damage, and underestimates it by 0.3 m^3^ (st) or 0.5% for 75% bark damage. These bark damage values resulted from measuring the degree of bark damage before and after stacking the logs according to the described methodology. HOPOSIM overestimates the net volume over bark of the real stacks for a bark damage of 21% by 0.4 m^3^ (st) or 1.2% and by 1 m^3^ (st) or 3.2% under bark. Regarding the net volume of the stacks, the comparison was made for 21% bark damage as this was the damaged bark degree given as all logs were measured by means of the xylometer.

**Table 1:** Basic descriptive statistics of the simulation results and performed measurements of real stacks.

Gross volume measured according to the RVR method in m^3^ (st)							
			Real stacks			Simulated stacks	
Bark damage percentage (%)			21	75		21	75
Avg.			49.0	46.7		49.2	46.44
Min.			47.4	45.4		46.5	44.16
Max.			50.3	48.2		52.0	49.59
Net volume for a 21 % bark damaged in m^3^							
		Real stacks			Simulated stacks (on average)		
Over bark	Avg.	34.4			34.8		
	Min.				34.7		
	Max.				34.8		
Under bark	Avg.	31.1			32.1		
	Min.				32.1		
	Max.				32.2		

Calculating the RMSE for simulations runs, and considering both bark damages, it could be shown that the deviation of the simulated gross volume from the measured gross volume can be quantified in a range from 0.7 m^3^ (st) to 2.1 m^3^ (st), with an average deviation of 1.2 m^3^ (st) or 2.4%, ranging from 1.4% to 4.2%. Furthermore, the computed MBE revealed that the simulation model underestimates the gross volume of the real stacks on average by -0.01 m^3^ (st), ranging from - 1.04 m^3^ (st) to 1.69 m^3^ (st), or by -0.02% on average, ranging from -2.1% to 3.4%.

**Fig 8:**
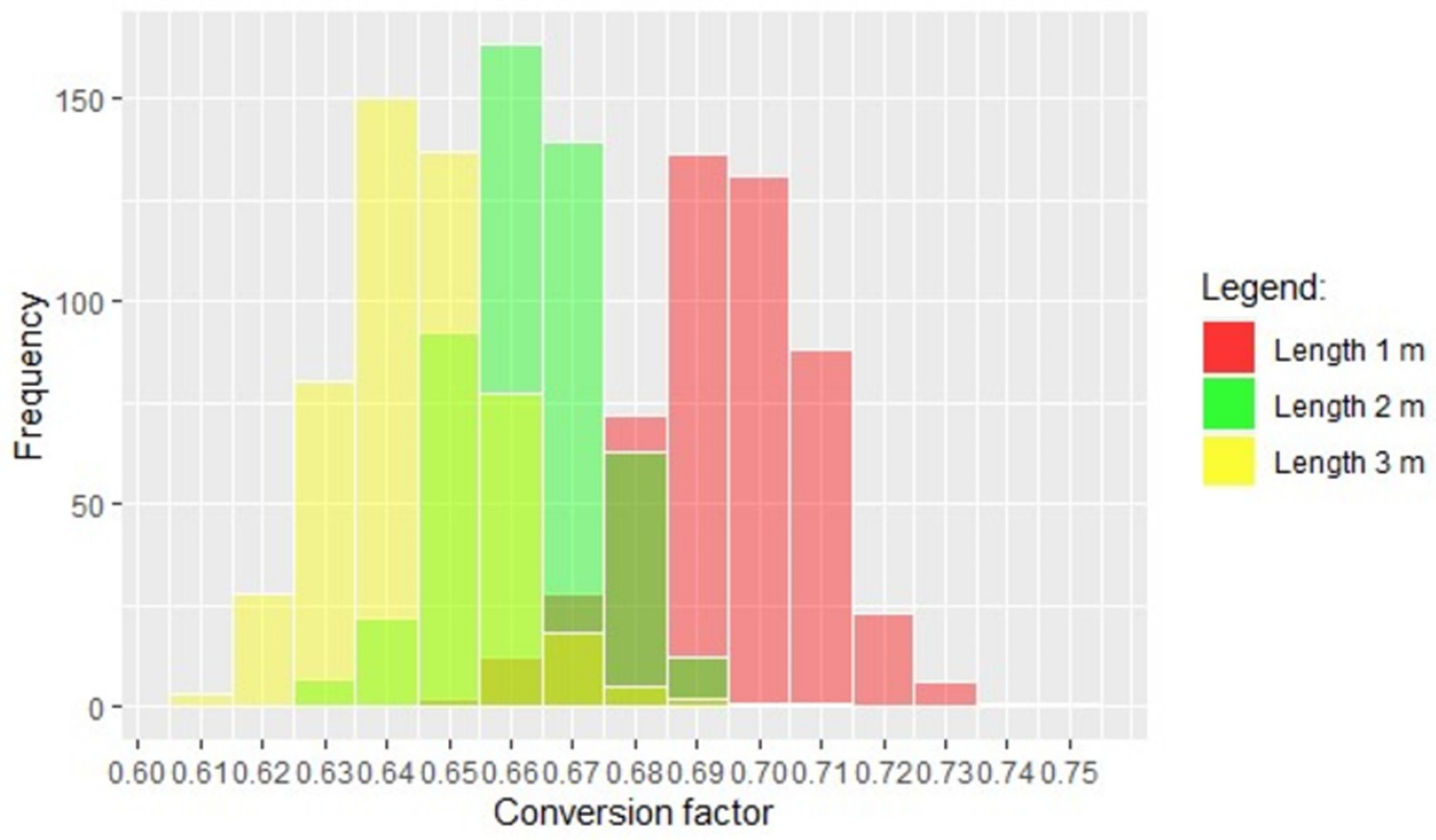
Distribution of the resulted conversion factors.

Finally, with the purpose of examining the sensitivity of the model, three simulation runs with 500 iterations, respectively, were carried out from different datasets. In each dataset, the parameter length was modified in a discretionary way. The other log parameters remained unaltered. The resulting conversion factors are displayed in Fig 8. In this histogram, the resulting conversion factors for log lengths of 3 m, 2 m and 1 m are depicted in yellow, green and red respectively.

In addition, the most frequent simulated conversion factors can be extracted. These are 0.64, 0.66 and 0.69 for the lengths of 3 m, 2 m and 1 m, respectively. Thus, an increasing trend can be observed when the length decreases.

Finally, it must be noted that to carry out each iteration, the simulation model needed 124 seconds on average, ranging from 108 to 137 seconds.

## Discussion

### Development of the simulation model

At present, HOPOSIM can be deemed a very powerful tool. From the simulation outcomes, several current research issues can be addressed, beginning with the aforementioned analysis of the individual influence of log and stack parameters on stack volume and conversion factors, to deduction of conversion factors for each wood assortment or timber type. HOPOSIM offers a broad spectrum of possibilities. It makes it possible to analyze the accuracy of different measurement methods for different wood assortments: RVR method versus photo-optical method. In addition, the use of the simulation model makes it possible to analyze the influence on the stack volume when measuring the gross volume from the stack´s two sides or from only one of them. Furthermore, the surface occupied by the logs´ ends and the surface of voids in the simulated stacks can be measured. From these data, it is possible to obtain results that contrast with the results from applying the quadrant method or to evaluate the stacking quality. Moreover, using HOPOSIM it is possible to quantify the bark portion in each simulated stack, which allows assessment of its economic value when commercializing the roundwood over bark. HOPOSIM also allows development of new measurement guidelines where the influence of all parameters is considered and quantified, considering specific assortments with reference to different tree species and according to the provenance regions. In this regard, current measurement guidelines, e.g., from Sweden, can be tested using the software HOPOSIM. Moreover, the measurement guidelines developed from the results provided by HOPOSIM make possible a prompt estimation of the solid wood content of a stack. From the simulation outcomes, the degree of influence of a determined parameter can be evaluated, and solid wood content can then be determined more efficiently using a quick visual assessment of the logs that constitute the stack [30]. Additionally, the simulation model could predict situations that have not yet been analyzed, e.g., importations of exotic roundwood, and provide the stakeholders reliable information in advance upon which future commercial operations can be based.

Nevertheless, although the quality of the simulation performance is very high and the logs and stacks could be simulated in detail, it must be noted that the simulation model has some limitations. In particular, it is not able to virtually reproduce burls or infrequent crookedness forms that can be found in the natural environment. Nor can it consider the particularities of the terrain. Under these circumstances, the model is not currently capable of providing accurate results.

Generally, at the moment, HOPOSIM is not a tool to provide the stakeholders of the roundwood supply chain with a quick, preliminary estimation regarding the total gross volume obtained from the wood harvest. For that purpose, other tools such as photo-optical methods in combination with conventional conversion factors might be more suitable. Still, HOPOSIM could become a powerful decision support tool for stakeholders in the near future. To achieve this improvement, the data introduction should be facilitated so that the desired results can be obtained directly from, e.g., TLS data or previous studies on tapering and crookedness values for each tree species. In doing so, it would not be necessary anymore to measure each log individually. Instead, locally well adapted default factors for feeding the model could be developed and would support to obtain reliable results at stand level.

### Exemplary model evaluation

Considering the comparison of simulation outputs with the measurements of gross volume and net volume over bark, it can be stated that the simulation model is capable of simulating wood stacks with a high degree of similarity to the real ones. Only a slight deviation from the volume of the measured stacks resulted for the net volume under bark. However, this deviation could possibly be caused by an inaccurate measurement of the bark thickness of the real logs, leading to an imprecise estimation of the bark volume in the real stacks. Nevertheless, the reduced deviation of the simulated gross volumes from the measured ones for both bark damage percentages implies that the simulation model can accurately reproduce the reduction of the bark volume when the log´s bark is damaged.

Regarding the evaluation approach, it must be pointed out that HOPOSIM is a stochastic simulation model, i.e., each stack is modelled randomly. Due to the numerous combinations that could be modelled, a mere comparison of the results of one simulation of a determined number of stacks with the same number of measured stacks cannot be accepted nor considered enough to judge the model accuracy. The likelihood that a modelled stack is simulated identically to a real stack in terms of the position of the logs is extremely low where there are few simulations. To tackle this issue, it was necessary to perform a statistically relevant number of simulations in order to compare their results with the real measurements. In doing so, it was possible to determine the minimum and maximal deviation of the simulation model with respect to the reality as well as the average deviation in absolute terms and percentages. Consequently, an efficient approach to evaluate this model would have been the virtual identical reproduction of the real stacks, where each log would have occupied the same position as in the real stacks. However, the random modelling process of HOPOSIM did not make this possible. Therefore, it could not be proven whether the resulting deviations were due to inaccuracy of the model or to the fact that virtual stacks identical to the real ones were not simulated within the total 1000 iterations performed. In this regard, it could also be possible that the limitations of the model described above could cause such marginal deviations.

With regard to the sensitivity analysis, the resulting conversion factors are similar to the conversion factors included in the measurement guidelines of several countries for this assortment, e.g., Netherlands [36] and Austria [13]. Beginning with the conversion factors included in the German RVR [3], the average simulated conversion factors deviated slightly, ranging from 1.4% for 1 m to 6.6% for 3 m, depending on the length. It must be pointed out that the conversion factors that appear in the RVR are not considered to be precise in the same document, being the result of previous statistical databases. In addition, these values are not classified for different assortments such as tree species but only for these different lengths. Therefore, a comprehensive comparison between the simulated conversion factors and the conversion factors included in the RVR was not possible.

Furthermore, the average simulated conversion factors are within the range described by Fonseca [9] in his compilation. In addition, the most frequent simulated conversion factors for lengths of 2 and 3 m coincide exactly with the conversion factors for the assortment destined for manufacture pallets included in the Dutch measurement guideline [36], deviating only for 1 m at 1.4%. Furthermore, the deviation from the conversion factors included in the Austrian guideline is 1.4% for a length of 1 m [13]. The average simulated conversion factors would be deviated from the conversion factors included in the Polish reference to 1.4% for every length of the log [14]. Moreover, the resulting decreased trend as the length increases coincides with the trend shown in each of the aforementioned measurement guidelines and the research works of Zon [37] and Câmpu *et al*. [12]. This is due to the effect of some log parameters such as the crookedness. Thus, when the logs are stacked, air spaces are produced that become larger the longer the logs are. In contrast, the shorter the logs are, the closer the logs can be stacked and the higher the solid wood content and the conversion factors will be [37].

Finally, the cost savings offered by the simulation model must be noted. During the data collection, the required time of each stacking process was measured. Whereas the simulation of each stack takes a short period of time, on average 124 seconds, the building process of each real stack required an average of 30 minutes, taking into consideration the favorable terrain conditions, which facilitated the stacking work of the forwarder and saved much work time. This demonstrates that the simulation model avoids the high costs which would have been necessary in the real world to collect the amount of data required to investigate the current research issues derived from a broad statistical basis.

## Conclusion

The simulation model integrated into software was developed successfully. As anticipated, the approach of using a simulation model makes it possible to obtain large databases that allow the investigation of the current research issues in this research field on a sufficient statistical basis. In this way, HOPOSIM solves the limitation of the high costs associated with collecting the required data in the field.

According to the calculated MBE, the simulation model negligibly underestimates the gross volume of the measured stacks and slightly overestimates the net volume over and under bark of the measured stacks. In addition, it could be proven that the model is capable of providing credible results when the input parameters are modified such as the length in this exemplary case. This makes it possible to analyze the influence that log and stack parameters have on the gross volume, net volume and conversion factors.

The influence of the parameters crookedness and taper on the stack volume was analyzed [30, 38]. In this way, the usefulness of this model in the scientific field has already been demonstrated.
